# Upregulating the Expression of LncRNA ANRIL Promotes Osteogenesis via the miR-7-5p/IGF-1R Axis in the Inflamed Periodontal Ligament Stem Cells

**DOI:** 10.3389/fcell.2021.604400

**Published:** 2021-02-22

**Authors:** Minxia Bian, Yan Yu, Yuzhi Li, Zhou Zhou, Xiao Wu, Xiaying Ye, Jinhua Yu

**Affiliations:** ^1^Institute of Stomatology, Nanjing Medical University, Nanjing, China; ^2^Key Laboratory of Oral Diseases of Jiangsu Province and Stomatological Institute of Nanjing Medical University, Nanjing, China; ^3^Endodontic Department, School of Stomatology, Nanjing Medical University, Nanjing, China

**Keywords:** lncRNA ANRIL, inflamed periodontal ligament stem cells, miR-7-5p, IGF-1R, differentiation

## Abstract

**Background:**

Long non-coding RNA (lncRNA) antisense non-coding RNA in the INK4 locus (ANRIL) is a base length of about 3.8 kb lncRNA, which plays an important role in several biological functions including cell proliferation, migration, and senescence. This study ascertained the role of lncRNA ANRIL in the senescence and osteogenic differentiation of inflamed periodontal ligament stem cells (iPDLSCs).

**Methods:**

Healthy periodontal ligament stem cells (hPDLSCs) and iPDLSCs were isolated from healthy/inflamed periodontal ligament tissues, respectively. The proliferation abilities were determined by CCK-8, EdU assay, and flow cytometry (FCM). The methods of Western blot assay (WB), quantitative real-time polymerase chain reaction (qRT-PCR), alizarin red staining, alkaline phosphatase (ALP) staining, ALP activity detection, and immunofluorescence staining were described to determine the biological influences of lncRNA ANRIL on iPDLSCs. Senescence-associated (SA)-β-galactosidase (gal) staining, Western blot analysis, and qRT-PCR were performed to determine cell senescence. Dual-luciferase reporter assays were conducted to confirm the binding of lncRNA ANRIL and miR-7-5-p, as well as miR-7-5p and insulin-like growth factor receptor (IGF-1R).

**Results:**

HPDLSCs and iPDLSCs were isolated and cultured successfully. LncRNA ANRIL and IGF-1R were declined, while miR-7-5p was upregulated in iPDLSCs compared with hPDLSCs. Overexpression of ANRIL enhanced the osteogenic protein expressions of OSX, RUNX2, ALP, and knocked down the aging protein expressions of p16, p21, p53. LncRNA ANRIL could promote the committed differentiation of iPDLSCs by sponging miR-7-5p. Upregulating miR-7-5p inhibited the osteogenic differentiation of iPDLSCs. Further analysis identified IGF-1R as a direct target of miR-7-5p. The direct binding of lncRNA ANRIL and miR-7-5p, miR-7-5p and the 3′-UTR of IGF-1R were verified by dual-luciferase reporter assay. Besides, rescue experiments showed that knockdown of miR-7-5p reversed the inhibitory effect of lncRNA ANRIL deficiency on osteogenesis of iPDLSCs.

**Conclusion:**

This study disclosed that lncRNA ANRIL promotes osteogenic differentiation of iPDLSCs by regulating the miR-7-5p/IGF-1R axis.

## Introduction

Periodontitis is a chronic infectious disease of periodontal support tissue, during which osteoclast activates and leads to bone resorption (Hienz et al., 2015). Until now, the reconstruction and repair of periodontal support tissue have been an unsolved problem in oral clinic treatment. Thus, periodontal tissue regeneration has become a hotspot of research in the region of oral medicine research in recent years. Increasing studies about periodontal ligament stem cells (PDLSCs) emerged as PDLSCs are considered to be the important seed cells of periodontal tissue regeneration and repair (An et al., 2016). PDLSCs are a type of tissue−specific mesenchymal stem cells (MSC) and have corresponding specialties, such as self−renewal, multipotency, immunosuppressive response. They can differentiate into chondrogenic, adipogenic, and osteogenic lineages *in vivo* and *in vitro* (Wada et al., 2009; Tsumanuma et al., 2011; Nunez et al., 2019). However, under the condition of inflammation, the osteogenic differentiation capacity is reduced along with the accelerated process of cell senescence of remaining iPDLSCs, and then iPDLSCs cannot achieve effective periodontal support tissue regeneration (Yi et al., 2017; Rotini et al., 2018). The methods to facilitate the directional differentiation ability of iPDLSCs might provide a new strategy for clinical treatment of periodontitis.

LncRNAs are a class of non-coding RNAs longer than 200 nt in length. They have received extensive attention as burgeoning regulators involved in diverse biological processes (Liu Y. et al., 2019). Huang et al. (2015) reported that lncRNA H19 may be involved in osteogenesis, invasion, and migration of human MSCs. As a kind of lncRNA with a base length of about 3.8 kb, lncRNA ANRIL has been discovered to play an important role in many cancers. A lot of studies revealed that lncRNA ANRIL could regulate cell growth or proliferation in several cancers including retinoblastoma, colorectal cancer, and cervical cancer (Naemura et al., 2016; Zhang J. J. et al., 2018; Wang X. et al., 2019). Besides, lncRNA ANRIL also takes part in the senescence of vascular smooth muscle cells (Tan et al., 2019). However, the effects of lncRNA ANRIL on osteogenesis and senescence of iPDLSCs and underlying mechanisms remain elusive.

MicroRNAs (miRNAs) are a highly conserved group of short non-coding RNAs found in most tissues, and they can regulate post-transcriptional gene expression (Lu and Rothenberg, 2018; Zhang J. J. et al., 2018). Meanwhile, miRNAs play an indispensable role in developmental and cellular processes such as metabolism (Rottiers and Näär, 2012), cell cycle (Bueno and Malumbres, 2011), differentiation (Ivey and Srivastava, 2010), and signal transduction (Inui et al., 2010). Increasing evidence has indicated that miRNAs are involved in multiple physical performances of iPDLSCs, including proliferation and differentiation (Li X. B. et al., 2018). Mir-7-5p is the most studied miRNA sequence in the microRNA-7(miR-7) family, which is a crucial miRNA that plays a variety of roles in physiological and pathological processes (Pogribny et al., 2010; Kalinowski et al., 2014). Studies have shown that miR-7-5p can inhibit cell proliferation, promote apoptosis by regulating the EGFR/Akt/mTOR and RelA/NF-κB signaling pathways and play the role of “tumor suppressor gene” in various malignant tumors (Giles et al., 2016; Song et al., 2016). By regulating the STAT3/miRNA-7-5p, the osteoblast capacity of MSCs can be influenced (Tang et al., 2020). Nevertheless, clarification of the role of miR-7-5p on the regulatory mechanism of osteogenic differentiation of iPDLSCs is still unclear.

Insulin-like growth factor-1 receptor (IGF-1R) is a pervasive growth receptor and has been found on the surface of many kinds of cells, including hepatocytes, myocytes, and osteocytes (Wang Z. et al., 2019; Zhang et al., 2019). It is also involved in the regulation of proliferation, apoptosis, differentiation, and malignant transformation of cancer cells (Zhang et al., 2019). Insulin-like growth factor (IGF-1) is the most affluent growth factor in the bone matrix, which can combine with IGF-1R to maintain bone mass and form new bone (Xian et al., 2012). Our previous studies demonstrated that IGF-1R has a crucial impact on the osteo/odontogenic differentiation of DPSCs and SCAPs (Shu et al., 2016; Liu et al., 2018). Similarly, numerous studies have shown that the activation of IGF-1/IGF-1R can regulate cell senescence and osteogenesis, participate in maintaining intracellular homeostasis, and promote tooth-related tissue regeneration (Sergi et al., 2019; Zhou et al., 2019). This study explores the role of lncRNA ANRIL/miR-7-5p/IGF-1R axis in the osteogenic differentiation of inflamed periodontal ligament stem cells for the first time.

## Materials and Methods

### Cell Culture

IPDLSCs were isolated from teeth with periodontitis from periodontitis patients (*n* = 20, aged 28–45 years) with their informed consent referred to the Jiangsu Provincial Stomatological Hospital. At the same time, the Medical Ethics Committee of the Stomatological School of Nanjing Medical University approved this study. The patients diagnosed with severe chronic periodontitis at active inflammatory stage and teeth needed to be removed were defined as alveolar bone loss no less than 2/3 and more than 1 pocket (depth 5 mm), and hPDLSCs of human healthy impacted third molars were acquired from patients aged from 28 to 45 years.

The teeth were washed thrice with phosphate-buffered saline (PBS, Gibco, Life Technologies, United States), then PDL was separated from the middle third of the root using a surgical scalpel, digested in medium containing 3 mg/ml collagenase type I (Sigma, St. Louis, MO, United States) and 4 mg/ml trypsin (Beyotime, Haimen, China) for 30 min at 37°C. After digestion, we centrifuged at 1,000 r/min for 5 min, and tissue clumps were collected. The hPDLSCs and iPDLSCs were grown in alpha minimum essential medium (α-MEM; Gibco, Life Technologies, United States) with 10% fetal bovine serum (FBS, Gibco, Life Technologies, United States), 100 mg/ml streptomycin, and 100 units/ml penicillin at 37°C in a humidified incubator containing 5% CO_2_. The medium was replaced every 3 days. When the cells grew to 70–80% confluence, trypsin was used to digest and the cells were collected for passage in a 1:3 radio. The cells from passage 3 to 5 were used for following experiments in the present study.

### Characterization and Differentiation Assay

To identify the phenotypes of hPDLSCs and iPDLSCs, the surface markers of mesenchymal stem cells were detected by flow cytometry. Cells at passage 3 were incubated with primary antibodies for human CD105, CD90, CD73, CD29, CD34, and CD45 (all from BD Pharmingen, San Diego, CA, United States) according to the manufacturer’s instructions. The incubation procedure was carried out at 4°C in the dark for 1 h, then cells were rinsed twice with PBS and subjected to flow cytometric analysis.

HPDLSCs and iPDLSCs were seeded in six-well plates with adipogenic induction medium and cultured in 15 ml conical−bottomed sterile tubes with chondrogenic induction medium, respectively. The cells were then cultured in a 5% CO_2_ incubator at 37°C for 25 days. The adipogenic medium was changed every 3 days, and the chondrogenic medium was changed every 2 days. After 25 days, the cells were analyzed for adipogenesis and chondrogenesis by Oil Red O staining and Alcian Blue staining. HPDLSCs and iPDLSCs were also seeded in six-well plates with osteogenic induction medium, and the cells were analyzed by Alizarin red staining after 14 day culturing. The stained cells were photographed using a microscope.

### Flow Cytometry

Cell cycle analysis was operated according to previous study’s protocol (Li et al., 2019). In brief, hPDLSCs and iPDLSCs were collected by trypsin and fixed with 70% cold ethanol overnight at 4°C in dark. Washed with PBS, samples were measured using FAC Scan flow cytometer (BD Biosciences, San Jose, CA) and independently analyzed three times.

### Western Blot Analysis

After being cultured for 7 days, transfected cells were rinsed with PBS two times and total protein was extracted using RIPA buffer (Beyotime) containing phenylmethylsulfonyl fluoride on ice. After centrifugation, proteins were collected and denatured by boiling for 10 min. Proteins were separated by 10% SDS-PAGE and transferred to 0.22 μm PVDF membranes (Millipore, Shanghai, China). After incubated with primary antibodies including OSX (ab22552), RUNX2 (ab76956), ALP (ab95462), IGF-1R (ab182406) from Abcam, P16 (#80772), P21 (#2947), P53 (#2527) from Cell Signaling Technology, GAPDH (Protein Tech Group) overnight at 4°C, the membranes were incubated with secondary antibodies for another 1 h at room temperature, followed by TBST wash for 30 min and 10 min each. The band density was quantified by Image J software.

### Quantitative Real-Time-Polymerase Chain Reaction (RT-PCR) Analysis

After 7 days of induction, total cellular RNA was extracted by Trizol reagent (Invitrogen, United States), and using a PrimeScript RT Master Mix kit (TaKaRa, Otsu, Japan) to reverse transcribe into cDNA according to conventional protocols. qRT−PCR was conducted using the ABI 7300 real−time PCR system. Triplicate reactions (20 μl volume) were performed. Two internal normalized controls including *GAPDH* and *U6* were used for mRNA and miRNAs, respectively. The expression levels of *LncRNA ANRIL*, *miR-7-5P*, *OSX*, *ALP*, *RUNX2*, *P16*, *P21*, *P53*, and *IGF-1R* were detected with qRT-PCR. The data were analyzed using the 2^−ΔΔCt^ relative expression method. The PCR primer sequences are listed in Table 1.

**TABLE 1:** Primer sequences.

Genes	Primes	Sequences (5′–3′)
*ANRIL*	Forward	CTAAGGAGCAGAAGACATC
	Reverse	GTAGAATCTCTCAGACGGTTG
*OSX*	Forward	CCTCCTCAGCTCACCTTCTC
	Reverse	GTTGGGAGCCCAAATAGAAA
*RUNX2*	Forward	TCTTAGAACAAATTCTGCCCTTT
	Reverse	TGCTTTGGTCTTGAAATCACA
*ALP*	Forward	ACCTGAGTGCCAGAGTGA
	Reverse	CTTCCTCCTTGTTGGGTT
*P16*	Forward	CCCCGATTGAAAGAACCAGAGAG
	Reverse	TACGGTAGTGGGGGAAGGCATA
*P21*	Forward	AGCGACCTTCCTCATCCACC
	Reverse	AAGACAACTACTCCCAGCCCCATA
*P53*	Forward	AGCTTTGAGGTGCGTGTTTGTG
	Reverse	TCTCCATCCAGTGGTTTCTTCTTTG
*IGF-1R*	Forward	AGGATATTGGGCTTTACAACCTG
	Reverse	GAGGTAACAGAGGTCAGCATTT-T
*GAPDH*	Forward	GAAGGTGAAGGTCGGAGTC
	Reverse	GAGATGGTGATGGGATTTC
*miR-7-5p*	Forward	CAGGGAGGCGTGGATCACTG
	Reverse	CGTCGGGGGCTCATGGAGCGG
*U6*	Forward	CTCGCTTCGGCAGCACA
	Reverse	AACGCTTCACGAATTTGCGT

### Immunofluorescence Staining

The transfected cells were inoculated into a 12-well plate with a coverslip in each hole and cultured in mineralized medium for 7 days. After washing with PBS twice, the cells were fixed with 4% paraformaldehyde for 30 min. The cells were then penetrated with 0.25% Triton-100 at room temperature for 12 min, washed several times with PBS, and sealed with goat serum (DCS/BioGenex, Hamburg, Germany) at 37°C for 45 min. The cells were then incubated with an antibody against RUNX2 and ALP at 4°C overnight, and then incubated with a fluorescence-labeled secondary antibody at room temperature for 45 min, and the nuclei were dyed with 4′,6-diamidino-2-phenylindole (DAPI; Beyotime) for 90 s. Images were observed using an inverted fluorescent microscope (Olympus, Shanghai, China).

### Cell Counting Kit-8 Assay and EdU

Cell proliferative activity was measured using the Cell Counting Kit-8 (CCK-8, Dojindo, Tokyo, Japan). Transfected cells were seeded in 96-well culture plates at a density of 2 × 10^3^ cells/well in complete culture medium. CCK-8 reagent (10 μl) and 90 μl of α-MEM were added to each well at the indicated time points (days 0, 1, 3, 5, 7, 9). After incubation for 2 h, the cells were assessed at 450 nm absorbance by a microplate reader.

Transfected iPDLSCs were inoculated on 12-well plates with 4 × 10^5^ per well and incubated with EdU medium for 4 h at 37°C. Then the cells were fixed with 4% paraformaldehyde for 30 min and incubated with 0.25% triton-100 for 10 min. After rinsing with PBS three times, 1 × Apollo staining solution was added to incubate for 30 min, and the DNA was stained with Hoechst 33342 for 20 min in darkness, subsequently observed under a fluorescence microscope and EdU analysis was quantified using Image J software.

### Alkaline Phosphatase Staining and Activity

At 7 days after transfected iPDLSCs were seeded in six-well plates and cultured in osteogenic medium, BCIP/NBT staining kit (Beyotime, China) was used to assess the osteogenic differentiation according to the instructions. The cells were washed with PBS and fixed with 4% paraformaldehyde for 30 min, then rinsed three times with PBS and the alkaline solution was added to each well. ALP quantitative analysis was performed using an alkaline phosphatase assay kit (Jian Cheng, Nanjing, China) following the protocol.

### Alizarin Red Staining

Cell mineralization was evaluated by ARS staining, and transfected iPDLSCs were cultured in osteogenic induction medium for 14 days. The cells were washed with PBS three times, and 4% paraformaldehyde was added to fix the cells for 30 min. The alizarin red solution was added, 1 ml/well, for 30 min at room temperature. Then the mineralized nodules were photographed by using an inverted microscope.

### Senescence-Associated β-Galactosidase Staining (SA-β-Gal)

The cellular senescence of transfected iPDLSCs was measured by SA-β-Gal staining kit (GenMed Scientifcs Inc., Shanghai, China). Briefly, the cells were washed with GENMED cleaning fluid, then covered with fixed fluid at room temperature for 5 min and incubated with SA-β-Gal staining solution at 37°C without CO_2_ for 24 h. After incubation, the sections were washed twice in PBS and mounted in glycerol and observed under a microscope. Image J software was used to quantify the senescent cells.

### Cell Transfection

LncRNA ANRIL overexpression and knockdown were conducted via lentiviral transfection. IPDLSCs were transfected into the following groups: NC group, ANRIL group, sh-NC group, and sh-ANRIL group. Recombinant lentiviruses were synthesized by Gene Chem (Shanghai, China). The miR-7-5p mimics and inhibitor transfected to overexpress and inhibit miR-7-5p in iPDLSCs were purchased from Ribobio (Guangzhou, China). The transfected cells were divided into groups: NC group, mimics group, iNC group, and inhibitor group.

### Fluorescence *in situ* Hybridization

Ribo^TM^ Fluorescent *in situ* Hybridization Kit (RiboBio) was used to perform FISH experiments according to the protocols. Briefly, the cells were grown on the slides, and when grown 60–70% confluence, we rinsed the cells with PBS and fixed them with 4% paraformaldehyde. Then, the cells were incubated with hybridization mixed with FISH probe overnight at 37°C in dark. After washing with washing buffer, the cells were counterstained with 4,6-diamidino-2-phenylindole and visualized using a confocal microscope.

### Dual-Luciferase Reporter Gene Assay

LncBase Predicted v.2 was performed to determine the binding sites between ANRIL and miR-7-5p, as well as potential targets of miR-7-5p and IGF-1R were predicted by TargetScan. MiR-7-5p mimics or normal control was co-transfected with IGF-1R-MUT, IGF-1R-WT, lncRNA ANRIL-MUT, or lncRNA ANRIL-WT into 293T cells following the manufacturer’s instructions. Luciferase activity was detected using a Dual-Luciferase Reporter Assay System (Promega, Madison, United States).

### Statistical Analysis

All consequences were presented as the mean and standard deviation (mean ± SD). and the experiments were performed in triplicates. GraphPad Prism 5.0 software and SPSS 20.0 software were utilized for statistical analyses. Statistical significance was established at *P* < 0.05.

## Results

### Characterization of Periodontal Ligament Stem Cells

PDLSCs were successfully isolated from the collected healthy teeth and the teeth with periodontitis, respectively. The adherent cells had spindle-like morphology (Supplementary Figures 1A, 2A). Flow cytometry assay described that these cultured cells were positive expressions of CD29, CD73, CD90, and CD105, and hardly express CD34 and CD45 (Supplementary Figures 1C, 2C). The results illustrated that the isolated cells were mesenchymal stem cells. Chondrogenic and adipogenic differentiation was tested by Alcian Blue staining and Oil Red O staining. At the same time, osteogenic differentiation was verified by Alizarin Red Staining (Supplementary Figures 1D, 2D). Furthermore, Supplementary Figures 1B, 2B showed that MSC surface molecule STRO-1 was observed on the cell surface of the periodontal ligament stem cells.

### Long Non-coding RNA (LncRNA) Antisense Non-coding RNA INK4 Locus Is Down-Regulated in Inflamed Periodontal Ligament Stem Cells Compared With Healthy Periodontal Ligament Stem Cells

**FIGURE 1:**
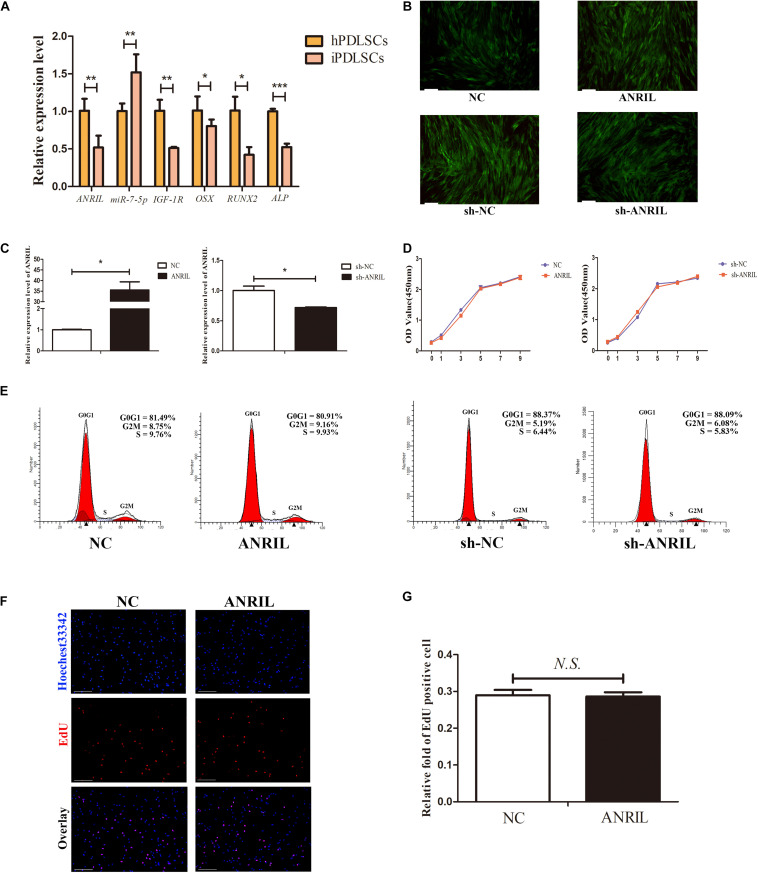
**(A)** The expression levels of long non-coding RNA (*lncRNA) antisense non-coding RNA INK4 locus (ANRIL),miR-7-5p, insulin-like growth factor-1 receptor (IGF-1R), alkaline phosphatase (ALP), OSX*, and *RUNX2* were determined by quantitative real-time polymerase chain reaction (qRT-PCR) in healthy periodontal ligament stem cells (hPDLSCs) and inflamed periodontal ligament stem cells (iPDLSCs). **(B)** Fluorescent photomicrographs showed lentivirus transfection in NC, ANRIL, sh-NC, sh-ANRIL groups. (Scale bar = 100 μm). **(C)** The transfection efficacy of ANRIL was measured by qRT-PCR in ANRIL group and sh-ANRIL group. GAPDH was used for normalization. **(D)** Proliferative ability of iPDLSCs was detected by Cell Counting Kit-8 (CCK-8) assay. **(E–G)** Flow cytometry and EdU assay demonstrated that ANRIL had no significant difference in cell proliferation of iPDLSCs on day 7 (Scale Bar = 100 μm; N.S., *P* > 0.05) (*P < 0.05, **P < 0.01, ***P < 0.001).

HPDLSCs and iPDLSCs were cultured in complete medium. We performed qRT-PCR to assess the expression of lncRNA ANRIL, miR-7-5p, IGF-1R, ALP, OSX, and RUNX2 in hPDLSCs and iPDLSCs. We found that expressions of ANRIL, IGF-1R, ALP, OSX, and RUNX2 were significantly decreased while miR-7-5p was increased in iPDLSCs compared with hPDLSCs (Figure 1A) (*P* < 0.05). The results indicated that iPDLSCs had lower osteogenic activity compared with hPDLSCs, and lncRNA ANRIL might play a positive role in osteogenic differentiation potential.

### Long Non-coding RNA Antisense Non-coding RNA INK4 Does Not Markedly Affect the Proliferative Ability of Inflamed Periodontal Ligament Stem Cells

To explore the vital role of lncRNA ANRIL in iPDLSCs, we first studied the impact on the proliferative ability of iPDLSCs *in vitro*. Cells were transfected with lentivirus and transfection efficacy was confirmed by qRT-PCR analysis (Figures 1B,C) (*P* < 0.05). CCK-8 assay indicated that lncRNA ANRIL had no significant difference in cell viability between ANRIL-group and NC-group (*P* > 0.05), and sh-ANRIL group and sh-NC group also showed the same result (Figure 1D). Meanwhile, flow cytometry analysis revealed that the proliferation index (PI = G2M + S) has no statistical significance in the ANRIL group and NC group as well as in the sh-ANRIL group and sh-NC group (Figure 1E). The above conclusions were further confirmed by EdU experiment (Figures 1F,G).

### Long Non-coding RNA Antisense Non-coding RNA INK4 Locus Affects the Osteogenesis and Senescence of Inflamed Periodontal Ligament Stem Cells

**FIGURE 2:**
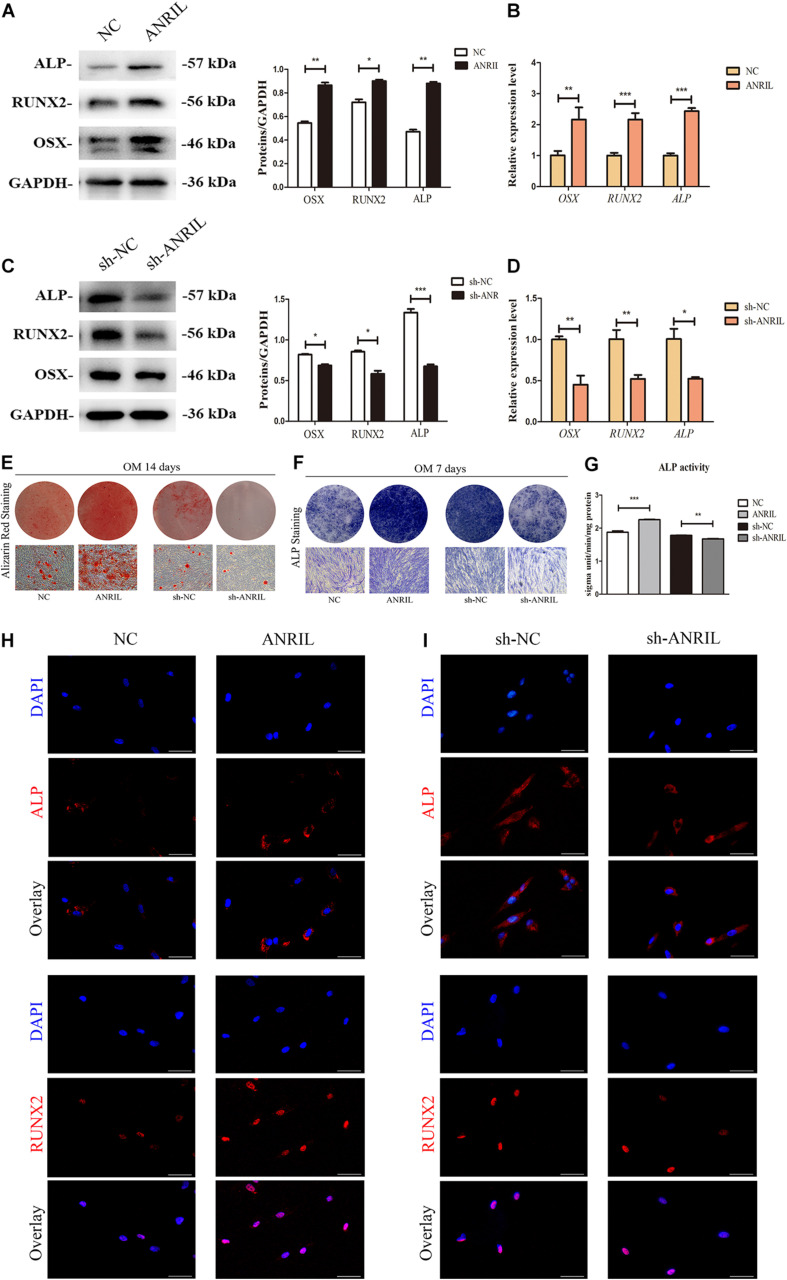
LncRNA ANRIL regulates osteogenic differentiation of iPDLSCs. **(A–D)** ALP/*ALP*, RUNX2/*RUNX2*, and OSX/*OSX* expressions were measured by western blot and qRT-PCR on day 7 after osteogenic induction. GAPDH served as an internal control. **(E–G)** Osteogenic differentiation of iPDLSCs was determined by Alizarin Red S, ALP staining and ALP activity assay at 14 and 7 days after osteogenic induction. **(H,I)** The expressions of ALP and RUNX2 in transfected iPDLSCs were also determined by immunofluorescence assay (Scale Bar = 50 μm) (*P < 0.05, **P < 0.01, ***P < 0.001).

The cells were transfected with lentivirus and transfection efficacy was confirmed by qRT-PCR (Supplementary Figure 3A) (*P* < 0.05). Cultured with mineralized induced medium for 7 days, western blot confirmed that the expression levels of osteogenic differentiation proteins (ALP, RUNX2, and OSX) were significantly up-regulated, and qRT-PCR analysis further revealed that lncRNA ANRIL overexpression significantly increased *ALP*, *OSX*, *RUNX2* mRNA levels (Figures 2A,B) (*P* < 0.05). The above results suggested that overexpression of lncRNA ANRIL promoted osteogenic differentiation of iPDLSCs. ALP activity, ALP staining, alizarin red staining, and immunofluorescence staining further confirmed the above conclusion (Figures 2E–H). On the contrary, sh-ANRIL group showed that down-regulating lncRNA ANRIL could inhibit osteogenesis the opposite trend (Figures 2C,D,G,I). These results indicated that lncRNA ANRIL enhanced the osteogenic differentiation of iPDLSCs.

**FIGURE 3:**
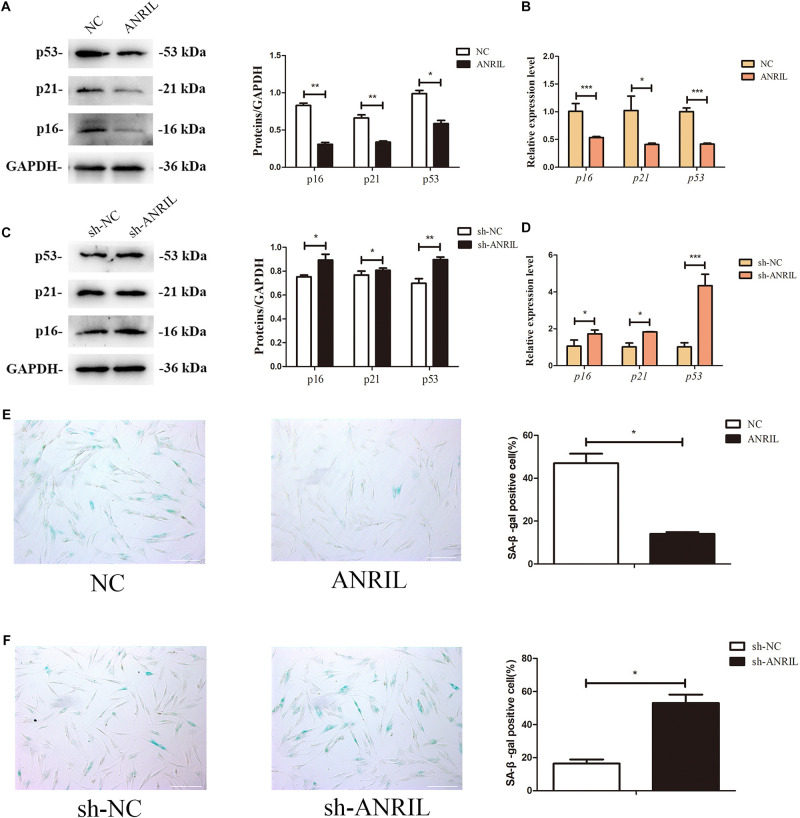
LncRNA ANRIL affects the senescence of iPDLSCs. **(A)** Protein expression of p16, p21, and p53 in the ANRIL group and NC group. **(B)** mRNA levels of *p16*, *p21*, and *p53* in the ANRIL group and NC group. **(C)** Protein expression of p16, p21, and p53 in the sh-ANRIL group and sh-NC group. **(D)** mRNA levels of *p16*, *p21*, and *p53* in the sh-ANRIL group and sh-NC group. **(E,F)** SA-β-gal staining showed less senescent cells in the ANRIL group and less senescent cells in the sh-ANRIL group. Scale bar = 100 μm (*P < 0.05, **P < 0.01, ***P < 0.001).

Then we investigated the effects of lncRNA ANRIL on cell senescence of iPDLSCs. After 7 days cultured in complete culture medium, the protein levels of p16, p21, and p53, as well as mRNA expression of *p16*, *p21*, and *p53* were downregulated in the ANRIL group while upregulated in the sh-ANRIL group as shown in Figures 3A–D (*P* < 0.05). At the same time, the positive expression of SA-β-gal in ANRIL group was significantly decreased compared with NC group, while it was increased in the sh-ANRIL group compared with the sh-NC group (Figures 3E,F). Thus, our data suggested that lncRNA ANRIL postponed the cell senescence of iPDLSCs.

### MiR-7-5p Is the Direct Target of Long Non-coding Antisense Non-coding RNA INK4 Locus

**FIGURE 4:**
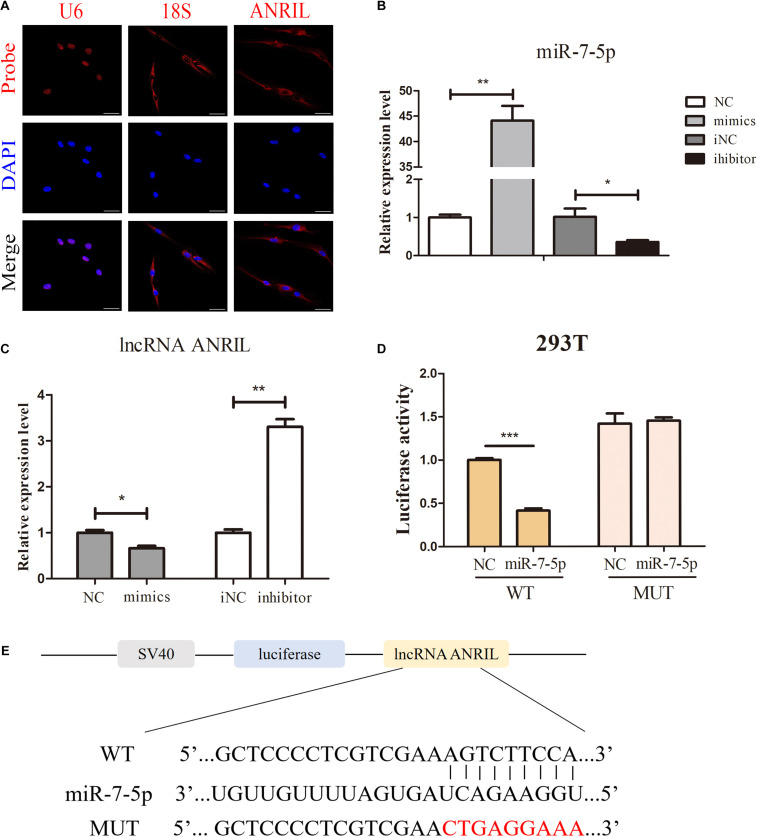
LncRNA ANRIL acts as a sponge of miR-7-5p. **(A)** FISH assay showed the localization of lncRNA ANRIL in the cytoplasm. 18S and U6 were the internal control. **(B)** Transfection efficacy of miR-7-5p mimics and inhibitor was determined by qPCR. **(C)** Relative lncRNA ANRIL expression level was measured by qPCR in iPDLSCs after transfected with miR-7-5p mimics or miR-7-5p inhibitor. **(C)** FISH assay showed the localization of lncRNA ANRIL in the cytoplasm. 18S and U6 were the internal control. **(D,E)** The binding of miR-7-5p to ANRIL was validated by conducting the luciferase reporter assay. IPDLSCs were co-transfected with mimics-NC or miR-7-5p mimics and wild-type lncRNA ANRIL or mutant lncRNA ANRIL. Relative Renilla luciferase activity was normalized to that of firefly luciferase. Scale bar = 100 μm (*P < 0.05, **P < 0.01, ***P < 0.001).

To study the molecular mechanism of how lncRNA ANRIL regulate the osteogenic differentiation of iPDLSCs, existing studies have shown that lncRNAs can act as a ceRNA or sponge miRNAs to regulate gene expression (Zhang L. M. et al., 2018; Li et al., 2020). To determine whether lncRNA ANRIL plays a regulatory role through the ceRNA mechanism, we first assured its localization in the iPDLSCs, and FISH results showed that lncRNA ANRIL was distributed in the cytoplasm (Figure 4A). Then we used LncBase Predicted v.2 in DIANA tools to analyze the predicted targets of lncRNA ANRIL, and we identified a lot of potential targets including miR-181a, miR-122-5p, miR-144, miR-125a, miR-7-5p, and so on. Among them, studies have shown that miR-7-5p plays a critical regulatory role in bone differentiation of MSCs. Thus, miR-7-5p was chosen for further study (Chen et al., 2020; Tang et al., 2020).

To investigate the relationship between miR-7-5p and lncRNA ANRIL, iPDLSCs were transfected with miR-7-5p mimics, miR-7-5p inhibitor, and correspond to miR-NC. Transfection efficacy results showed approximately 43-fold upregulation of miR-7-5p in the mimics group and 0.3-fold miR-7-5p expression in miR-7-5p inhibitor-transfected cells (Figure 4B). qRT-PCR results revealed that lncRNA ANRIL expression was reduced in the mimics group and elevated in the inhibitor group (Figure 4C) (*P* < 0.05). Moreover, dual-luciferase reporter assay uncovered that miR-7-5p mimics could specifically lessen only the luciferase activity of wild-type ANRIL but not miR−NC, while there was no apparent difference between the mutant ANRIL + miR-7-5p mimics group which further identified the miR-7-5p was a binding target of lncRNA ANRIL (Figures 4D,E) (*P* < 0.05). In general, the results above demonstrated the direct binding effect between lncRNA ANRIL and miR-7-5p in iPDLSCs.

### MiR-7-5p Inhibits the Osteogenic Differentiation of Inflamed Periodontal Ligament Stem Cells

**FIGURE 5:**
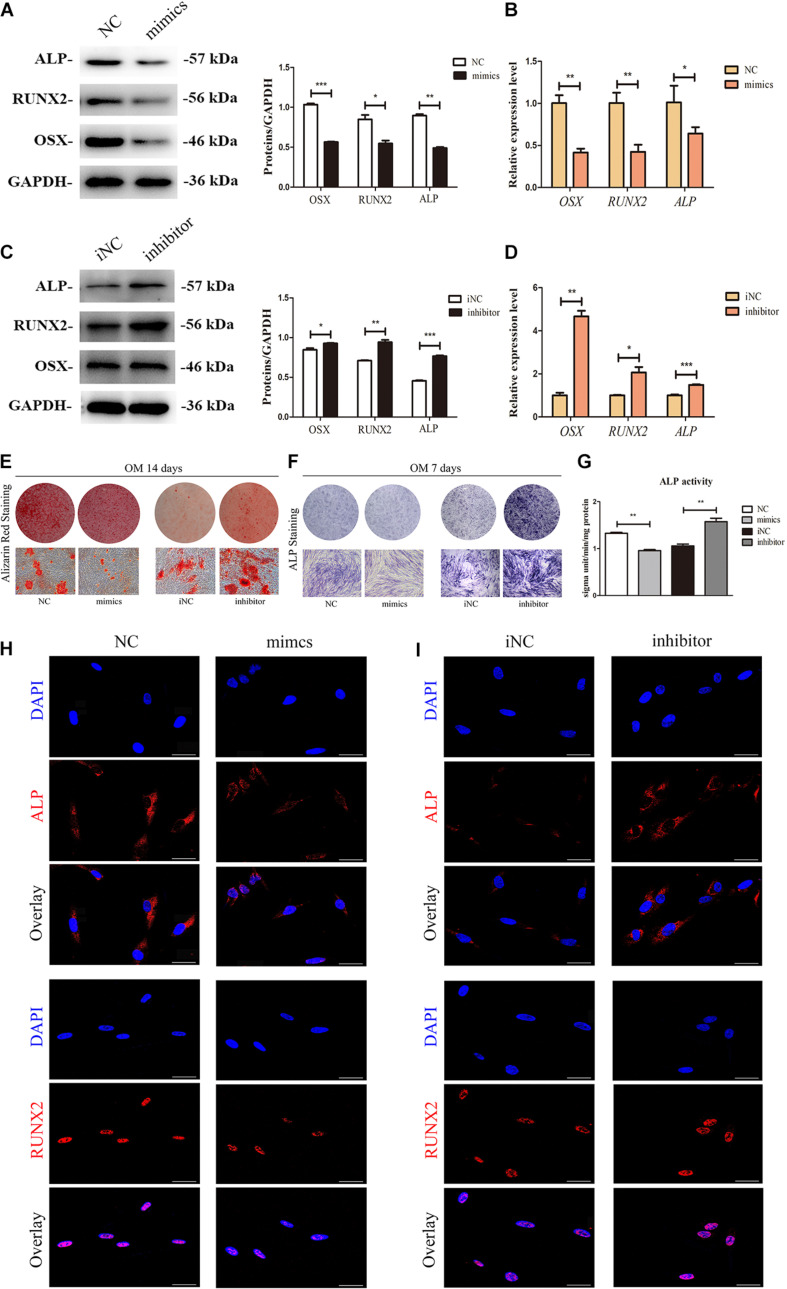
MiR-7-5p participates in osteogenic differentiation of iPDLSCs. **(A)** Western blot assay showed higher protein levels of ALP, RUNX2, and OSX in mimics group than the control group. GAPDH was the internal control. **(B)** qRT-PCR analysis of *RUNX2, OSX*, and *ALP* levels of iPDLSCs in control group and mimics group. **(C)** Western blot assay showed lower protein levels of ALP, RUNX2, and OSX in inhibitor group than the control group. GAPDH was the internal control. **(D)** qRT-PCR analysis of *RUNX2, OSX*, and *ALP* levels of iPDLSCs in control group and inhibitor group. **(E)** After 14 days of culture, Alizarin red staining showed more calcified nodules in the mimics NC group than mimics group and less calcified nodules in the inhibitor NC group than inhibitor group. **(F,G)** The ALP staining assay and ALP activity at Day 7 in the NC group, mimics group, iNC group and inhibitor group. **(H,I)** Immunofluorescence assay revealed downregulated ALP and RUNX2 in mimics group compared with NC group and upregulated ALP and RUNX2 in inhibitor group than iNC group. Scale bar = 100 μm (*P < 0.05, **P < 0.01, ***P < 0.001).

Cells were transfected with miR-7-5p mimics, miR-7-5p inhibitor, and correspond miR-NC; and transfection efficacy was confirmed by qRT-PCR (Supplementary Figure 3B) (*P* < 0.05). As shown in Figures 5A,C, expression of ALP, RUNX2, and OSX was significantly decreased in the mimics group, while increased in the inhibitor group on day 7. qRT-PCR also showed the same trend (Figures 5B,D) (*P* < 0.05). ARS, ALP activity, and ALP staining further verified that miR-7-5p inhibitor could promote the formation of mineralized nodules, whereas the miR-7-5p mimics impaired these processes (Figures 5E–G). Identically, expression of ALP and RUNX2 were downregulated by miR-7-5p mimics and upregulated by miR-7-5p inhibitor in iPDLSCs as immunofluorescence revealed (Figures 5H,I). These results indicated that miR-7-5p inhibited the process of osteogenic differentiation of iPDLSCs.

### MiR-7-5p Downregulates Insulin-Like Growth Factor-1 Receptor Expression in Inflamed Periodontal Ligament Stem Cells

**FIGURE 6:**
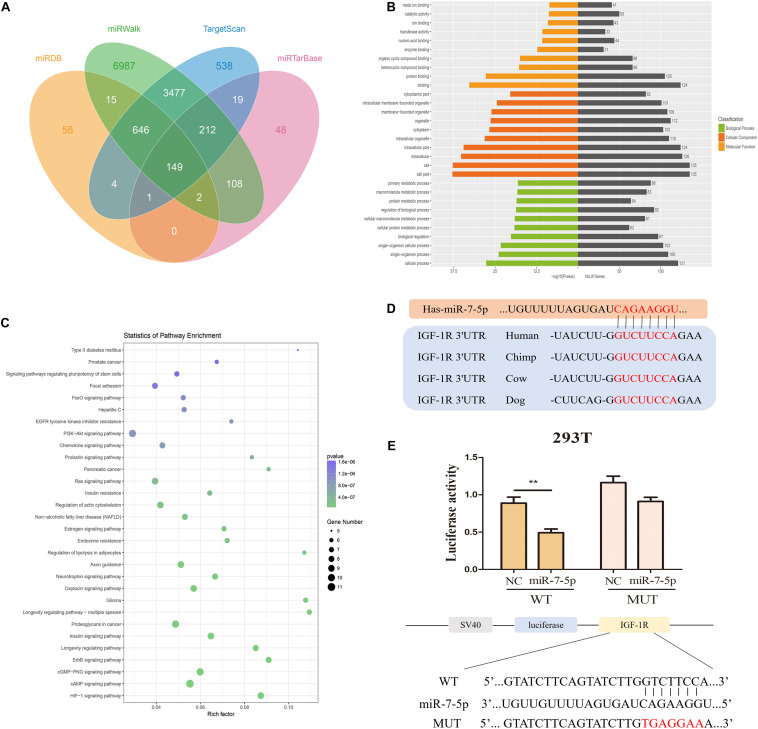
MiR-7-5p directly targets IGF-1R. **(A)** Venn diagram of four datasets (miRDB, miRTarBase, miRWalk, and TargetScan) showed miR-7-5p predicted target genes. **(B)** Gene Ontology (GO) is a commonly used approach for annotating genes and gene products with functions including molecular function, biological process, and cellular component. GO analysis showed the top 10 GO terms of each function which sorted by *P*-value. **(C)** Kyoto Encyclopedia of Genes and Genomes (KEGG) pathway analysis showed that target genes were enriched in regulation of significant signaling pathways. **(D)** The binding sequences between miR-7-5p and IGF-1R were predicted online. The red letters delegate the binding sequence of miR-7-5p. **(E)** Luciferase activity in 293T further illustrated that miR-7-5p could bind with IGF-1R (**P < 0.01).

Potential downstream target genes (12,264) of miR-7-5p were predicted according to bioinformatic analyses including miRTarBase, miRWalk, miRDB, and TargetScan algorithms (Figure 6A). GO annotation and KEGG pathway analysis suggested that these target genes participate in a variety of biological processes and cellular pathways, such as the PI3K-Akt signaling pathway (Figures 6B,C). However, most of these target genes which could play roles in iPDLSCs were not validated before. Among these genes, we found that IGF-1R is the mutual target gene of miR-7-5p in miRWalk, TargetScan, and miRTarBase databases. Furthermore, TargetScan 2.0 was performed to obtain the binding sites of miR-7-5p with IGF-1R, and the complementary regions were also highly conserved among different species (Figure 6D). Importantly, the positive regulatory effect of IGF-1R on the osteogenesis of tooth-derived stem cells had been documented in our previous studies (Shu et al., 2016; Liu et al., 2018).

Furthermore, dual-luciferase reporter assay showed no significant change in mutant-type IGF-1R group, but it was notably reduced in wild-type group, validating that miR-7-5p could bind with IGF-1R (Figure 6E). The above results proved that IGF-1R is a direct target of miR-7-5p.

### MiR-7-5p Inhibitor Rescues the Role of Long Non-coding RNA Antisense Non-coding RNA INK4 Locus Shortage on Inflamed Periodontal Ligament Stem Cells Osteogenesis

**FIGURE 7:**
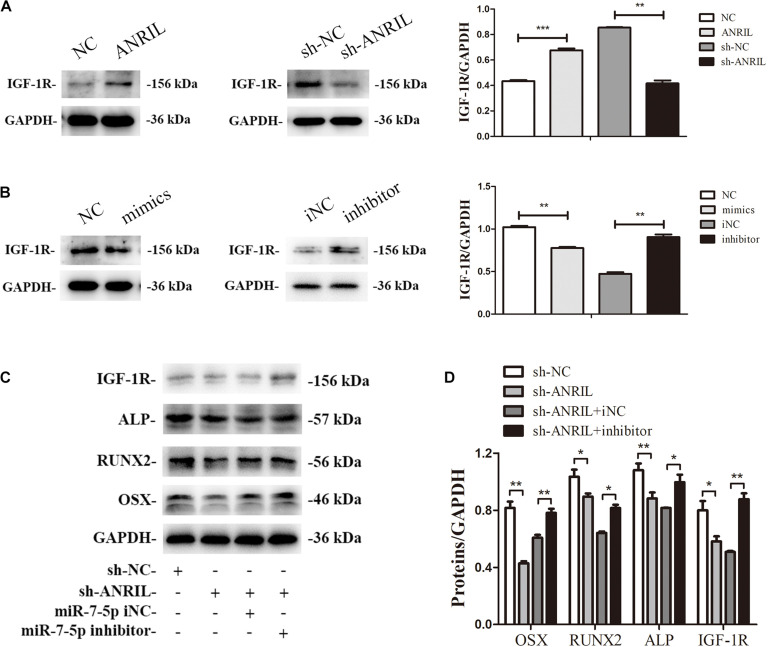
MiR-7-5p inhibitor rescues the role of ANRIL knockdown on committed differentiation of iPDLSCs. **(A)** IGF-1R expression in iPDLSCs transfected with overexpression and low-expression ANRIL. **(B)** IGF-1R expression in iPDLSCs transfected with miR-7-5p mimics, mimics NC, miR-7-5p inhibitor and inhibitor NC. **(C,D)** Expression of IGF-1R/*IGF-1R*, ALP/*ALP*, RUNX2/*RUNX2*, and OSX/*OSX* in iPDLSCs co-transfected with lncRNA ANRIL and miR-7-5p (*P < 0.05, **P < 0.01, ***P < 0.001).

To identify the interaction between miR-7-5p and lncRNA ANRIL and how they influence the IGF-1R in the osteogenic differentiation of iPDLSCs, we performed a rescue experiment in iPDLSCs transfected with sh-ANRIL and miR-7-5p inhibitor. The transfection efficacy was confirmed in Supplementary Figures 3C,D (*P* < 0.05). As presented in Figures 7A,B, Western blot proved that lncRNA ANRIL increased protein expression of IGF-1R and miR-7-5p suppressed IGF-1R in iPDLSCs. The Western blot showed that the expression of ALP, RUNX2, OSX, and IGF-1R declined in the sh-ANRIL group, while miR-7-5p inhibitor reversed the suppression of ANRIL knockdown on osteogenic differentiation of iPDLSCs (Figures 7C,D). In a word, lncRNA ANRIL absorbs miR-7-5p as a ceRNA and stimulates the IGF-1R to promote the committed differentiation of iPDLSCs.

## Discussion

Inflammation of the pathological periodontium in the periodontal pocket can change the cell biology of PDLSCs. Low-intensity chronic inflammation breaks the dynamic balance between pro-inflammatory and anti-inflammatory responses in the body. The body is in a state of inflammation for a long time, which accelerates the aging process and reduces the osteogenic differentiation of iPDLSCs. At the same time, aging also aggravates the state of inflammation, affects the resistance of patients to bacteria, and causing more severe and rapid destruction of periodontal tissues (An et al., 2018; Ebersole et al., 2018). Once it is damaged, the periodontium has a limited regeneration capacity, which relies on the availability of stem cells (Liu J. et al., 2019). Studies have shown that there are many other methods to regulate periodontal bone regeneration, such as bone grafts, scaffolds, and growth factors (Mahajan and Kedige, 2015). Our study focuses on the periodontal ligament stem cells in the inflammatory state to enhance osteogenic differentiation.

Recently, many studies have found that lncRNAs are not only associated with the occurrence and development of a variety of diseases but also with the committed differentiation of MSCs. For example, LncRNA H19 regulates the committed differentiation of SCAPs via miR-141/SPAG9 pathway (Li et al., 2019). Besides, LncRNA TUG1 facilitates osteogenic differentiation of PDLSCs by targeting Lin28A (He et al., 2018). LncRNA ANRIL was initially identified from patients with familial melanoma and encoded 3,834 nt RNA (Zhang J. J. et al., 2018). Jun-Jun et al. (2016). demonstrated that lncRNA ANRIL could inhibit the cell senescence of epithelial ovarian cancer. Moreover, lncRNA ANRIL markedly affects cell senescence of vascular smooth muscle cells directly sequesters miR-181a in the cytoplasm (Tan et al., 2019). However, the regulatory mechanisms of lncRNA ANRIL on osteogenic differentiation and senescence in periodontitis remain unclear.

In this study, we explored the expression of lncRNA ANRIL in iPDLSCs and hPDLSCs, and found that ANRIL was reduced in iPDLSCs. Studies have shown that periodontitis inhibits the formation of osteogenic, so we speculated that whether lncRNA ANRIL is associated with the osteogenesis of iPDLSCs (Peng et al., 2018). Here, we concluded that lncRNA ANRIL had no obvious effect on the proliferative ability of iPDLSCs. However, the influence of lncRNA ANRIL on cell proliferation was different in other experiments. For example, ANRIL could promote cell growth in head and neck squamous cell carcinoma (Matsunaga et al., 2019). Thus, we deduced that the effect of lncRNA ANRIL on cell proliferation might be different in various cell types. Herein, we checked the function of the lncRNA ANRIL in iPDLSCs osteogenic differentiation. The western blot and qRT-PCR showed that RUNX2/*RUNX2*, ALP/*ALP*, and OSX/*OSX* were increased, and aging indicators P53/*P53*, P21/*P21*, P16/*P16* were decreased when lncRNA ANRIL was overexpressed. It was confirmed that lncRNA ANRIL could promote the osteogenic differentiation and delay the senescence of iPDLSCs. Some studies have confirmed that osteogenic differentiation capacity decreases during the process of cell senescence. Therefore, we speculated the regulatory effects of lncRNA ANRIL on osteogenesis might be partly mediated by aging (Fan et al., 2018).

Many lncRNAs exert their miRNA sponge potential in multiple biological processes and compete for binding sites to affect the activity of targeted factors (Chen et al., 2017; Li et al., 2017; Tan et al., 2019). Increasing studies have shown that lncRNA ANRIL could be used as a ceRNA to regulate miRNAs expression and the activity of target genes. For example, lncRNA ANRIL promotes cell growth and represses apoptosis in retinoblastoma cells by targeting miR-99a (Wang X. et al., 2019).

MiR-7-5p has diverse roles in development and disease, and it may be an abnormal expression in different diseases. Emerging studies have confirmed that miR-7-5p acts as a tumor suppressor in various cancers including bladder cancer and pancreatic ductal adenocarcinoma (Li J. et al., 2018; Weihua et al., 2018). Meanwhile, miR-7-5p can play a regulatory role in MSCs osteogenesis (Chen et al., 2020; Tang et al., 2020). Nevertheless, the specific role of miR-7-5p in iPDLSCs has not been clarified yet. A previous study displayed that knockdown of lncRNA ANRIL could increase miR-7-5p expression in H9c2 cells (Shu et al., 2020). Given that, we assumed that lncRNA ANRIL also could function by binding with miR-7-5p in iPDLSCs. As expected, the sequence of lncRNA ANRIL binding with miR-7-5p was confirmed by bioinformatic analyses and dual-luciferase reporter gene assay. In this study, miR-7-5p knockdown silenced by a specific inhibitor could significantly promote osteogenesis, indicating the vital role of miR-7-5p in regulating the bone regeneration of periodontitis. We additionally found that the silence of miR-7-5p can attenuate the effects of ANRIL-knockdown on iPDLSCs, which further confirmed that ANRIL exerted its impacts on bone formation through sponging miR-7-5p. In this study, we indicated that miR-7-5p suppressed the osteogenic differentiation, which was the same as Li et al. confirmed, while Ta was different from cell types.

We studied the possible mechanism of how miR-7-5p regulates the osteogenesis of iPDLSCs. Bioinformatic analyses using miRDB, miRTarBase, miRWalk, and TargetScan algorithms have found that IGF-1R is a target gene of miR-7-5p. IGF-1R is a class of growth factor receptor proteins, which can play an essential role in the growth, proliferation, and differentiation of biological cells by promoting the protein synthesis process (Martin et al., 2019). Moreover, IGF-1R can mediate the biological actions of IGFs, which undergo autophosphorylation and thereby initiates cellular signaling cascades upon ligand binding. On the surface of periodontal ligament-derived fibroblasts, researchers discovered the presence of IGF-1R and an increase in IGF-1R expression during osteogenic differentiation of these cells (Reichenmiller et al., 2004; Gotz et al., 2006). Our previous studies indicated that the IGF-1/IGF-1R/*hsa-let-7c* axis affects the biological properties of dental stem cells by Tang et al. demonstrated that miR-7-5p might promote the osteoblastic differentiation of MSCs (Li X. B. et al., 2018; Tang et al., 2020). MiR-7-5p has an opposite effect on osteogenesis, which may be activating JNK and p38 MAPK pathways (Shu et al., 2016; Liu et al., 2018). Sergi et al. (2019) also found that following the ligand binding, the activated IGF-1 or insulin receptor can lead to the activation of two major signaling pathways, which are the MAPK pathway and the PI3K-PKB/AKT pathway. However, how IGF-1R plays a role in the senescence of iPDLSCs is still a question worthy of further study.

## Conclusion

To conclude, in this research we found that lncRNA ANRIL promotes bone formation by regulating miR-7-5p/IGF-1R, which further confirms the role of lncRNA ANRIL in bone formation.

## Data Availability Statement

The original contributions presented in the study are included in the article/Supplementary Material, further inquiries can be directed to the corresponding author/s.

## Ethics Statement

The studies involving human participants were reviewed and approved by the Medical Ethics Committee of the Stomatological School of Nanjing Medical University. The patients/participants provided their written informed consent to participate in this study. Written informed consent was obtained from the individual(s) for the publication of any potentially identifiable images or data included in this article.

## Author Contributions

MB, YY, and YL conducted the project design and the experiments, and wrote the manuscript. ZZ, XW, and XY performed the data analysis and reviewed the data. JY conceived and designed the study, and provided financial support and study materials. All authors read and approved the manuscript.

## Conflict of Interest

The authors declare that the research was conducted in the absence of any commercial or financial relationships that could be construed as a potential conflict of interest.

## Supplementary Material

Supplementary Figure 1 Phenotype identification of hPDLSCs. **(A)** The morphology of second-generation hPDLSCs. **(B)** Immunofluorescence assay showed that cultured hPDLSCs were positive for STRO-1. **(C)** The expression of CD29, CD73, CD90, CD105, CD34, and CD45 was detected by flow cytometry analysis. **(D)** Tri−lineage differentiation of hPDLSCs was performed *in vitro*. Scale bar = 100 μm.

Supplementary Figure 2 Phenotype identification of iPDLSCs. **(A)** The morphology of second-generation iPDLSCs. **(B)** Immunofluorescence assay revealed that cultured iPDLSCs were positive for STRO-1. **(C)** Flow cytometry analysis showed that iPDLSCs were positive for CD29, CD73, CD90, and CD105, and negative for CD34 and CD45. **(D)** Tri−lineage differentiation of IPDLSCs was performed *in vitro*. Scale bar = 100 μm.

Supplementary Figure 3 **(A)** The transfection efficacy of ANRIL was measured by qRT-PCR. **(B)** The transfection efficacy of miR-7-5p mimics and inhibitor was determined by qPCR. **(C)** The transfection efficacy of ANRIL was measured by qRT-PCR. **(D)** The transfection efficacy of miR-7-5p mimics and inhibitor was determined by qPCR (^∗^*P* < 0.05, ^∗∗^*P* < 0.01, ^∗∗∗^*P* < 0.001).
