# Supplementary figures and images for: Upregulating the Expression of LncRNA ANRIL Promotes Osteogenesis via the miR-7-5p/IGF-1R Axis in the Inflamed Periodontal Ligament Stem Cells
Source: Front Cell Dev Biol. 2021 Feb 22;9:604400. doi: 10.3389/fcell.2021.604400 (PMC7937634; doi:10.3389/fcell.2021.604400)

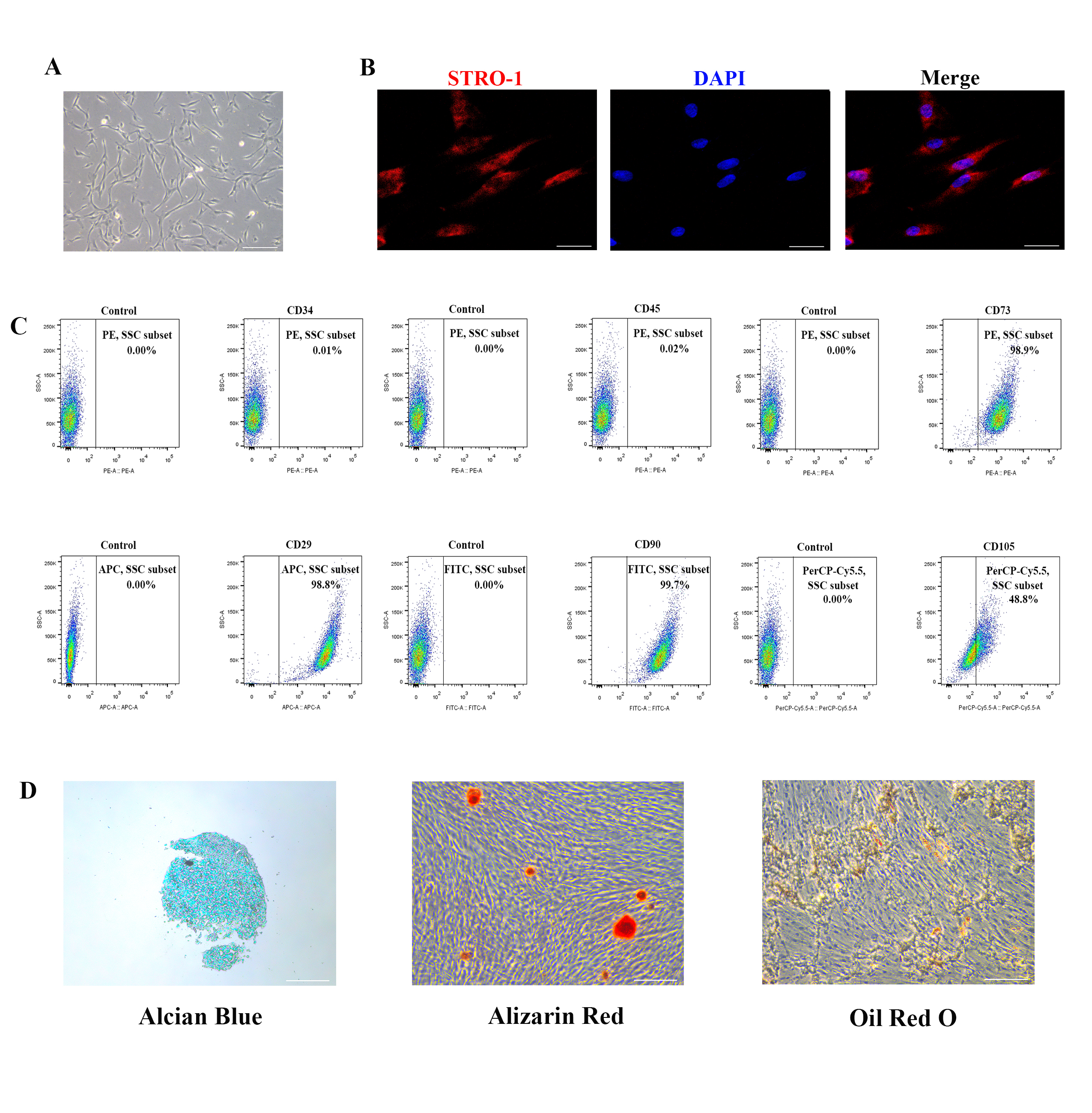

Supplement: Supplementary Figure 1 — Phenotype identification of hPDLSCs. (A) The morphology of second-generation hPDLSCs. (B) Immunofluorescence assay showed that cultured hPDLSCs were positive for STRO-1. (C) The expression of CD29, CD73, CD90, CD105, CD34, and CD45 was detected by flow cytometry analysis. (D) Tri−lineage differentiation of hPDLSCs was performed in vitro. Scale bar = 100 μm. [file Image_1.JPEG]

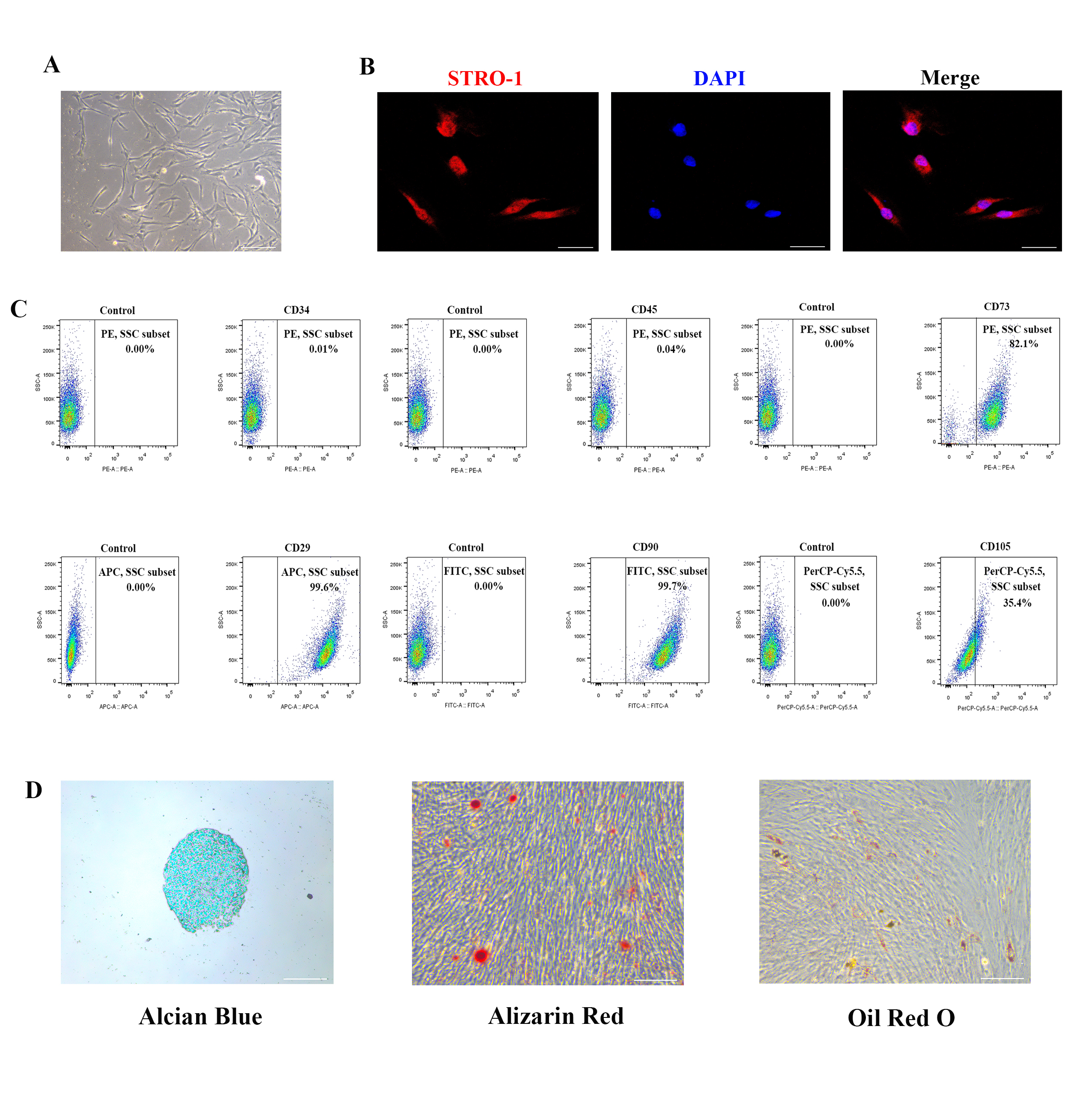

Supplement: Supplementary Figure 2 — Phenotype identification of iPDLSCs. (A) The morphology of second-generation iPDLSCs. (B) Immunofluorescence assay revealed that cultured iPDLSCs were positive for STRO-1. (C) Flow cytometry analysis showed that iPDLSCs were positive for CD29, CD73, CD90, and CD105, and negative for CD34 and CD45. (D) Tri−lineage differentiation of IPDLSCs was performed in vitro. Scale bar = 100 μm. [file Image_2.JPEG]

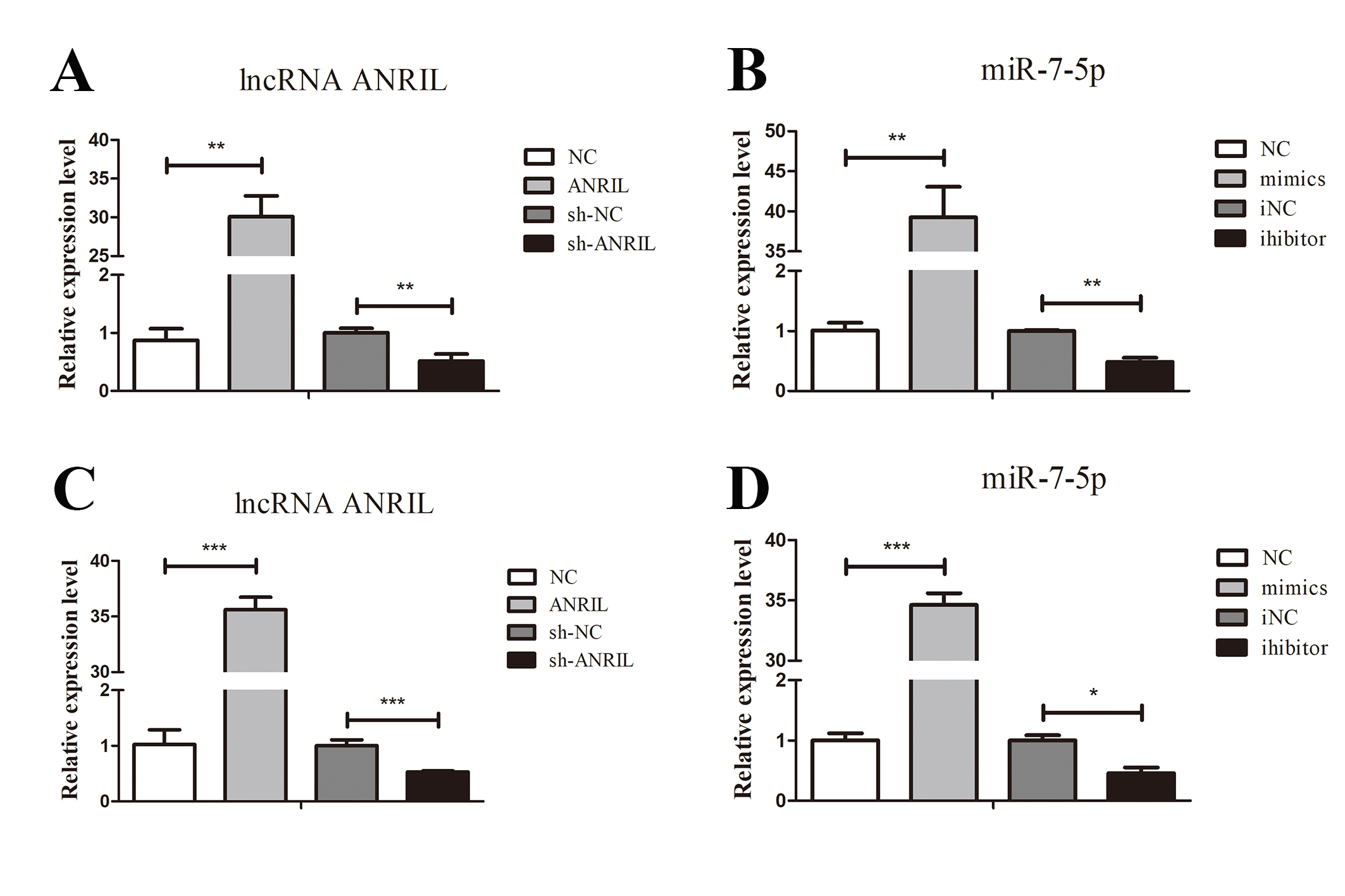

Supplement: Supplementary Figure 3 — (A) The transfection efficacy of ANRIL was measured by qRT-PCR. (B) The transfection efficacy of miR-7-5p mimics and inhibitor was determined by qPCR. (C) The transfection efficacy of ANRIL was measured by qRT-PCR. (D) The transfection efficacy of miR-7-5p mimics and inhibitor was determined by qPCR (∗P < 0.05, ∗∗P < 0.01, ∗∗∗P < 0.001). [file Image_3.JPEG]
